# Navigating AI: A Quick Start Guide for Healthcare Professionals

**DOI:** 10.7759/cureus.72501

**Published:** 2024-10-27

**Authors:** Piyush Mathur, Hajra Arshad, Rachel Grasfield, Reem Khatib, Avneep Aggarwal, Moises Auron, Avneesh Khare

**Affiliations:** 1 Anesthesiology, Cleveland Clinic, Cleveland, USA; 2 Research, BrainXAI ReSearch, BrainX LLC., Cleveland, USA; 3 General Medicine, Aga Khan Medical College, Karachi, PAK; 4 Anesthesiology, University of Iowa, Iowa City, USA; 5 Internal Medicine-Pediatrics, Cleveland Clinic, Cleveland, USA; 6 Anesthesiology, BrainXAI Research, BrainX LLC., Cleveland, USA

**Keywords:** artificial intelligence, checklist, computer programming, deep learning, machine learning

## Abstract

The rapid and nimble growth of artificial intelligence (AI) in healthcare has generated significant excitement among healthcare professionals. The most common question asked by clinicians about AI therefore is: “How do I get started?”. We outline a strategic approach for clinicians to integrate AI into their knowledge base, focusing on goal setting, creating a learning roadmap, identifying essential resources, and establishing success metrics. We have outlined practical steps, including acquiring programming skills and utilizing low-code platforms, based on an individual’s goal. Additionally, we present a resource toolkit that emphasizes continuous learning, collaboration, and mentorship to successfully adopt and implement AI in healthcare. We highlight the importance of understanding AI fundamentals and provide a roadmap to navigate a successful start.

## Introduction and background

Research and literature related to artificial intelligence (AI) in healthcare have seen exponential growth in recent years [1]. This trend is not just in the volume but also in the increase of qualitatively “mature” publications that demonstrate the evolution of AI in addressing complex healthcare challenges [1,2]. Most of the research and publications in the past have focused on image-based data and deep learning models, led by a few image-focused healthcare specialties such as Radiology, Gastroenterology, Pathology, Oncology, and Cardiology [1]. Research related to AI is evolving, as several other healthcare specialties broaden their focus to include the use of generative AI models [1,3]. The use of generative AI and multimodal models expands the opportunities to solve some of the most tenacious problems in healthcare.

With the growing adoption of AI in healthcare, there is an immense opportunity for clinicians to participate in all phases of research, development, evaluation, and its implementation. In a recent systematic review on the inclusion of clinicians in the development and evaluation of clinical AI tools, researchers found that developers consulted clinicians at various stages of AI algorithm development. Most of these consultations occurred inconsistently and were at the later stages of the AI algorithm design process (82%, 19/24 design studies) [4]. Only 33%(15 out of 45) of publications in the same study reported on subsequent clinician trust in the developed AI algorithms. This occurs despite the development of road maps provided by AI scientists which recommend having an inclusive multidisciplinary team approach from conceptualization to implementation of the AI algorithms in healthcare [5]. Considering that most of the AI algorithms are labeled as clinician decision support tools, concerns of burnout, bias, trust, and liability are real issues that need to be addressed [6,7]. Hence, clinician engagement and leadership in the development of these state-of-the-art solutions is vital for the successful adoption of AI in healthcare.

Understanding the basics of how AI models are built facilitates effective collaboration with technical teams, enhances decision-making regarding AI tools, and promotes advocacy for both patient care and clinicians’ operational needs. Learning about different aspects of AI algorithm development and subsequent implementation in clinical practice provides unique professional development opportunities for clinicians. A crucial element to discuss is the need to develop specific competencies for clinicians to effectively implement and utilize AI in their daily clinical practice [8].

Getting started with AI is a major roadblock for clinicians. While many resources exist that demystify AI for clinicians, there has been an ever-growing need to provide guidance on how to get started with AI for clinicians [9-11]. We propose an approach for clinicians to get started with AI involving four key steps goal setting, creating a roadmap, identifying resources, and measures of success.

## Review

Goal setting

Establishing goals for beginning the learning journey for AI in healthcare is a fundamental first step. AI is a continuously evolving and nimble science. Hence, just like any other scientific field, commitment to this domain for continuous learning and adoption is required. Having a formal learning plan and dedicated time to follow key learning resources with a specific goal is imperative.

The ability to read and understand research and publications related to AI in healthcare is elemental [12]. This requires developing an understanding of the key glossary related to AI, opportunities for application, basics of mathematical methods related to the models, basics of model architecture, and lastly, measures of performance of the models [12]. Understanding the differences between some of the key models, such as those using regression, classification, decision trees clustering, and neural networks, is important from the point of decision-making, to decide on their applicability toward specific problems. It is not just enough to run a few different models and use the one with the highest accuracy for application to a healthcare problem. Hence, it is essential for clinicians to understand the process of development of AI models and follow proposed pathways [13]. Understanding the relevance of how and why the model provided output is crucial, as a random selection of models may not guarantee the reproducibility of results.

Learning about the current state of publications in AI can help determine the key trends, successful methods, and hypotheses that have already been researched [14]. At the same time, this also might provide creative opportunities to think of the application of models to different fields of healthcare specialty which may have similar data and problems. Many healthcare specialties such as Gastroenterology and Pathology have leapfrogged in their research and development, learning from the use of convolutional neural networks (CNN) for image analysis previously utilized by Radiology and Ophthalmology [1]. As an example, CNN-based models embedded in endoscopes are being trialed and used by Gastroenterologists for colonoscopies [15]. As with any field of science, learning by hands-on experimentation provides much deeper insights into the problem and the desired solution. Practical development of AI solutions does require one to learn computer programming and have a higher grade of understanding of mathematical modeling techniques. Iterating over the problem and solution provides the continuous improvement approach to hone desirable skills. In a team environment, developing a solution programmatically may also provide an opportunity to receive feedback and develop a deeper understanding of the science [16].

**Figure 1:**
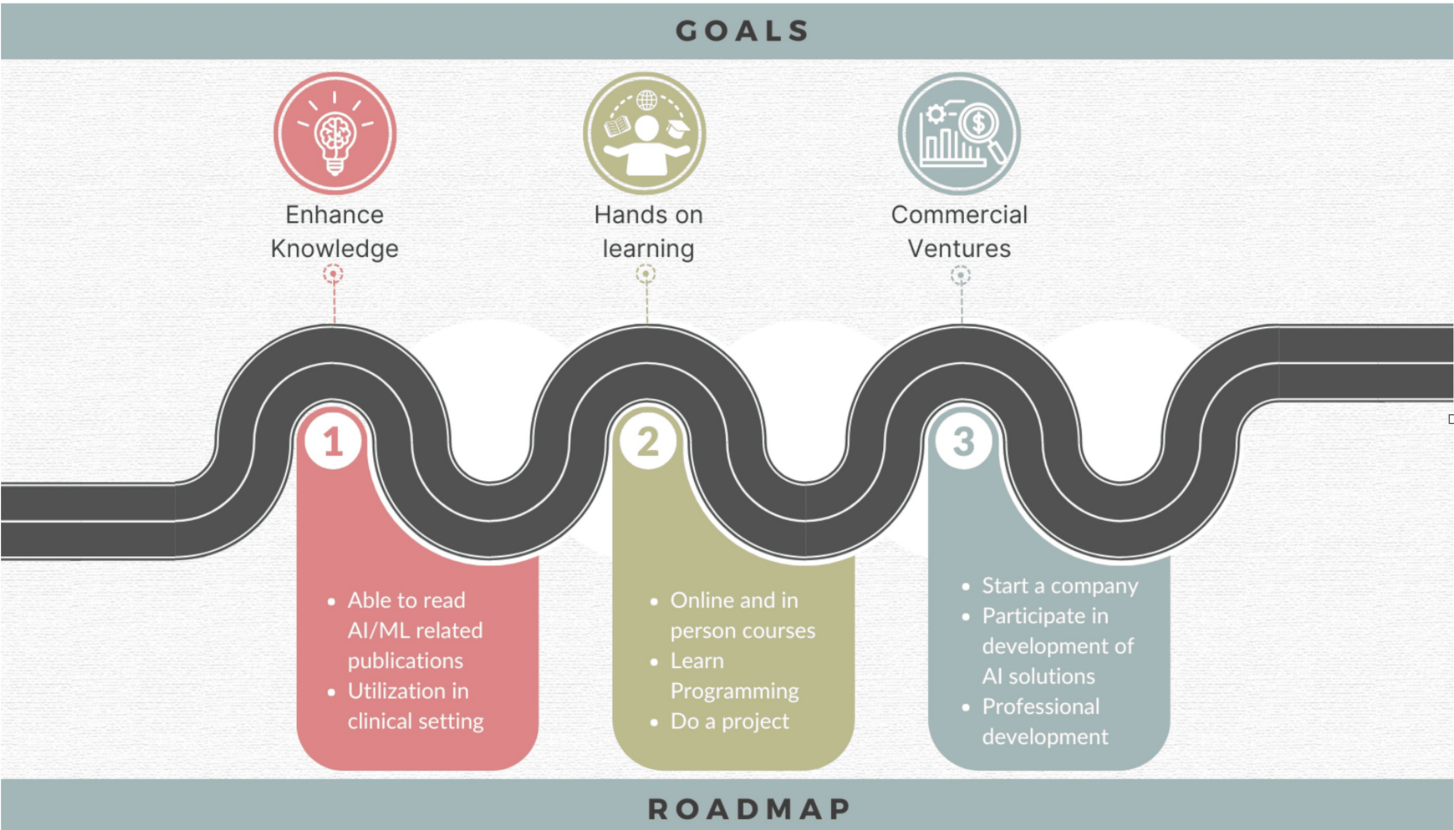
Setting goals and developing a personalized roadmap for AI in healthcare AI: artificial intelligence; ML: machine learning Image Credits: Hajra Arshad

Many clinicians are excited about the opportunities to develop and implement a commercially viable AI solution in healthcare. This requires a significant understanding of the basics of AI and an entrepreneurial approach [17]. One may not need to understand high-grade mathematics or AI to develop a commercial solution, however, a team of key members who are experts in the various domains including healthcare specialists, business leaders, data scientists, AI experts, system architects, developers, and application engineers, is necessary [18]. Setting goals and a personalized roadmap helps clinicians thereby navigate their learning or development path successfully (Figure 1).

The roadmap

By incorporating formal education about AI technology into medical curricula, the adoption and implementation of AI in healthcare can be greatly enhanced [19]. Formal training in programming languages like Python, akin to Point-of-Care Ultrasound (POCUS) training integration, can provide future clinicians with the skills they need to implement AI in healthcare. Although such training is beneficial to experienced clinicians as well, its incorporation into medical education can produce a new generation of healthcare workers capable of spearheading innovation and skillfully applying AI tools to enhance patient care [20]. As we advocate for the integration of AI education into formal medical curricula, it is imperative that clinicians take the initiative and actively participate in learning opportunities to bridge the gap until formal training becomes widely available [9].

Programming abilities, while not essential for all physicians, are extremely useful in the field of healthcare AI. Enrolling in a formal coding program has two key benefits: learning how to develop by tackling issues from a coding stance, and getting exposed to excellent programming techniques. The required level of technical proficiency in programming depends on whether a physician’s role is that of being an informed consumer, a translator, or a developer [11]. Nonetheless, being aware of the fundamentals of AI model construction makes it easier to work with technical teams, improves judgment when choosing AI tools, and encourages advocacy for both patient care and clinicians' operational needs [18].

Before participating in programming, clinicians should consider their interests, professional goals, and the specific needs of their clinical practice (Table 1).

**Table 1:** Pros and cons of learning computer programming for clinicians

Pros	Cons
Enhanced understanding of AI tools	Time-consuming
Improved collaboration with tech teams	Not directly clinically relevant
Opens innovative research opportunities	Steep learning curve
Ability to customize AI tools	Risk of information overload
Career advancement and diversification	Rapidly evolving field that requires continuous learning

Clinicians who want to learn programming might benefit from adopting a structured approach [21,22]. They should start by identifying current computing skills and establishing specific learning objectives. The next step is to select a programming language. Usually, Python is a good choice because of its easy syntax and applicability to machine learning projects. Enrolling in courses on basic programming helps by focusing on the foundational principles of programming. After that, one should practice coding on a daily basis utilizing tools like Kaggle, Jupyter Notebooks, and Google Colab. Cutting-edge tools such as GitHub Copilot and ChatGPT Plus, which offer AI-powered code suggestions and tutoring enhance the learning experience by improving interactivity and efficiency in the teaching process.

As skill levels increase, one may progress to more sophisticated topics like object-oriented programming and libraries. Understanding fundamental concepts such as linear algebra and statistics is also important because these mathematical concepts are required to manage data manipulation and optimize algorithms in machine learning applications. Support and networking opportunities can be utilized by participating in online forums, joining communities, and attending hands-on workshops. Advanced projects further improve learning, particularly those that call for interdisciplinary collaboration. Eventually, applying these skills in clinical settings will reveal ways in which programming can improve medical procedures. Maintaining a perpetual learning mindset and staying current with the latest developments is critical for long-term growth and programming proficiency.

Low-code/no-code platforms such as Orange Data Mining, KNIME, etc. provide useful options for clinicians who want to work with AI without having to do a lot of programming [23]. Using these platforms, clinicians can quickly create AI models without the need for complex coding knowledge [23]. However, they may not be ideal for advanced AI applications, which need more sophisticated programming.

Regarding formal courses, it may be useful to note that they often provide structured learning, which can aid in better comprehension. They save time and ensure that a solid foundation is built in a methodical manner, which is especially beneficial for beginners. Certifications that can help with professional development are often included. Furthermore, formal mentoring and coaching provide an excellent platform for enhancing the learning experience.

When selecting courses, clinicians must consider several critical factors to ensure the best possible learning experience. First and foremost, the credibility of the source is critical; choosing courses offered by reputable institutions or recognized platforms ensures quality education and valid certification. Second, relevance to healthcare is critical; courses should focus on AI and programming applications in the healthcare sector to provide the most applicable knowledge. Additionally, clinicians should avoid redundancy by being cautious of overlapping content across multiple courses, which can lead to redundant learning without meaningful progression. Finally, practical application is essential; clinicians should look for courses that emphasize real-world applications, as this is critical for translating theoretical knowledge into practical skills in clinical settings. Online courses are usually more flexible and accessible, often at a lower cost, whereas in-person courses offer direct interactions and hands-on support.

While courses are a good place to start, clinicians must engage in continuous learning using a variety of resources. Regular interaction with thought leaders, publications, podcasts, and professional communities allows them to stay current on the latest technologies and emerging trends. Participating in datathons is another excellent way to gain experience with data science in a collaborative setting [16]. The majority of these events require no prior knowledge of AI, making them accessible to non-technical participants. Clinicians can contribute their domain expertise to the team and acquire experience with AI terminology and implementation in a fast-paced challenge. These events can also spark project ideas that the team can pilot and develop further, providing practical, hands-on experience and collaborative learning [24]. Thus, understanding the differences in benefits and challenges between online and in-person courses is critical for making informed decisions (Table 2).

**Table 2:** Online vs in-person AI in healthcare course selection for clinicians

Aspect	Online Courses	In-Person Courses
Flexibility & Convenience	High (Learn anytime, anywhere)	Low (Fixed schedule and location)
Cost-Effectiveness	Generally more cost-effective	Higher costs (commuting, on-campus expenses)
Networking Opportunities	Access to global community, diverse networking	Direct peer interactions, immediate networking
Accessibility	Highly accessible; offers asynchronous learning	Requires physical presence, less accessible
Learning Style Suitability	Suited for self-motivated learners comfortable with technology	Ideal for hands-on learners requiring direct support
Structure and Discipline	Requires self-discipline due to flexible nature	Structured environment helps maintain discipline

Healthcare AI resource toolkit

**Figure 2:**
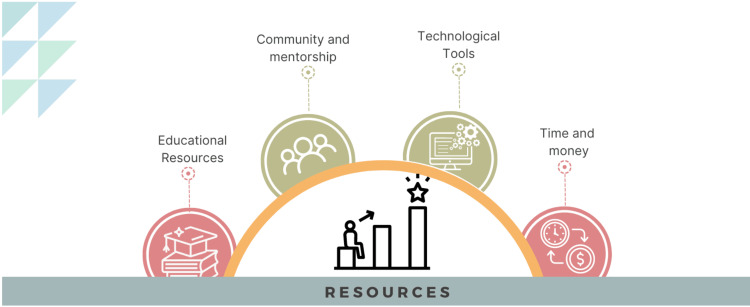
Identifying key resources to support AI in healthcare learning and development Image Credits: Hajra Arshad

The resource toolkit offers valuable information on various aspects critical for clinicians looking to learn about AI in healthcare. It includes a basic guide to educational resources available, and the technological resources needed by clinicians to facilitate their learning (Figure 2). Moreover, it emphasizes the value of peer support and mentoring, encouraging participation in professional associations and seeking advice from seasoned mentors. Time and financial considerations are also covered, providing clinicians with information about the required commitment and helping them to properly plan their learning path.

A variety of online courses and certifications are available for clinicians interested in AI, with options ranging from beginner to advanced (Table 3). These platforms provide flexible learning schedules and a variety of course options to accommodate different learning styles and objectives. Textbooks and academic journals provide in-depth knowledge and theoretical foundations for AI concepts and methodologies, making them valuable resources for those seeking to improve their understanding of specific AI topics (Table 3). Furthermore, physical events such as workshops, datathons, and conferences enable attendees to learn from experts, network with peers, and gain knowledge of the most recent advances and applications of AI in healthcare. These events offer practical learning opportunities, interactive sessions, and exposure to real-world AI projects.

**Table 3:** Learning resources for AI in healthcare

Online Courses and Certifications	Resource Link
American Board of AI in Medicine (ABAIM)	https://abaim.org [25]
AI in Healthcare Specialization (Coursera, Stanford Online)	https://www.coursera.org/specializations/ai-healthcare [26]
AI for Medicine Specialization (deeplearning.ai)	https://www.deeplearning.ai/courses/ai-for-medicine-specialization/ [27]
AI for Healthcare	https://www.ai4healthcare.org/[28]
Journals	
NEJM AI Journal	https://ai.nejm.org/ [29]
The Lancet Digital Health	https://www.thelancet.com/journals/landig/home [30]
NPJ Digital Medicine	https://nature.com/npjdigitalmed// [31]
Journal of American Medical Informatics Association(JAMIA)	https://academic.oup.com/jamia [32]
PLOS Digital Health	https://journals.plos.org/digitalhealth/ [33]
Books	
Translational Application of Artificial Intelligence in Healthcare	Translational Application of Artificial Intelligence in Healthcare: - A Textbook. (2023). United States: CRC Press [34]
AI in Clinical Medicine: A Practical Guide for Healthcare Professionals	AI in Clinical Medicine: A Practical Guide for Healthcare Professionals. (2023). United Kingdom: Wiley [35]
Hands-On Machine Learning with Scikit-Learn, Keras, and TensorFlow	Géron, A. (2019). Hands-On Machine Learning with Scikit-Learn, Keras, and TensorFlow: Concepts, Tools, and Techniques to Build Intelligent Systems. United States: O'Reilly Media [36]
Intelligence-Based Medicine: Artificial Intelligence and Human Cognition in Clinical Medicine and Healthcare	Chang, A. C. (2020). Intelligence-Based Medicine: Artificial Intelligence and Human Cognition in Clinical Medicine and Healthcare, Elsevier Science [37]
Strategies for Artificial Intelligence in Healthcare	Papay F., Mathur P., Mangus J. (2024). Strategies for Artificial Intelligence in Healthcare. Amazon (Kindle Direct Publishing) [38]
Data Sources	
Stanford AIMI	https://aimi.stanford.edu/shared-datasets [39]
Kaggle Datasets	https://www.kaggle.com/datasets [40]
Physionet	https://physionet.org/about/database/ [41]
UK Biobank	https://www.ukbiobank.ac.uk/enable-your-research/about-our-data [42]
BrainX Community Data	https://brainxai.org/data/ [43]
Harvard Dataverse	https://dataverse.harvard.edu/ [44]

Software tools and platforms that facilitate data preprocessing, model building, training, evaluation, and deployment are essential technological tools for clinicians interested in AI development and implementation. These tools may be chosen based on the AI project’s requirements and complexity. Also, compatible hardware resources and access to relevant, high-quality healthcare data must be available. Connecting with professional networks and organizations focused on AI in healthcare can give crucial support, knowledge sharing, and cooperation opportunities. Clinicians can interact with peers, mentors, and experts to share ideas, seek advice, and stay current on the newest developments. Finding a mentor with experience in healthcare AI can significantly accelerate the learning process and provide tailored guidance. Furthermore, participation in collaborative AI initiatives enables clinicians to obtain practical experience, learn from others, and contribute to vital healthcare solutions. Collaboration encourages teamwork, interdisciplinary learning, and the development of practical AI abilities.

Gaining proficiency in AI requires a significant time commitment, especially for clinicians with busy schedules. Balancing AI learning with clinical obligations requires effective time management and prioritization. Time must be set aside for learning and practicing with AI resources. This could be a set number of hours per week or spare time during breaks or commutes. Financial issues are also essential, as attending AI classes and using AI technologies can be costly. Online courses, certificates, workshops, and conferences can be costly, and obtaining appropriate software, hardware, or cloud computing resources may necessitate a financial investment. Clinicians should carefully weigh the cost-benefit ratio and look for potential financial or institutional support to help them on their AI journey.

Measuring success

**Figure 3:**
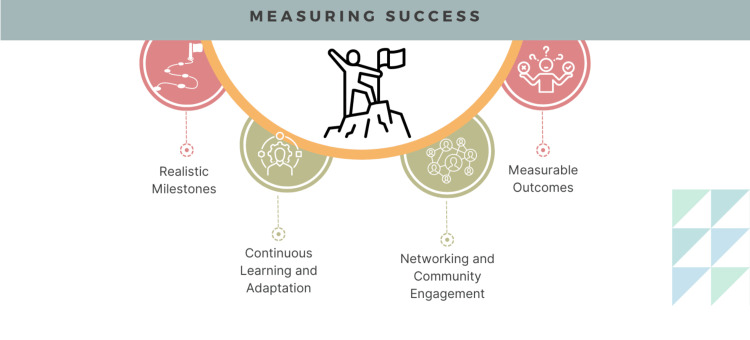
Developing measures of success for AI in healthcare journey Image Credits: Hajra Arshad

Milestones for success can be set in simple terms as creating a list of learning resources, developing a project plan outline, or starting with a pilot project to trial a concept (Figure 3). A strategy to define a roadmap to fulfill long-term aspirational milestones can be developed as one gets started with the short-term milestones and develops a strategy for success. Clearly defined metrics which could include some process measures or outcome measures are good to define as the roadmap is created.

**Figure 4:**
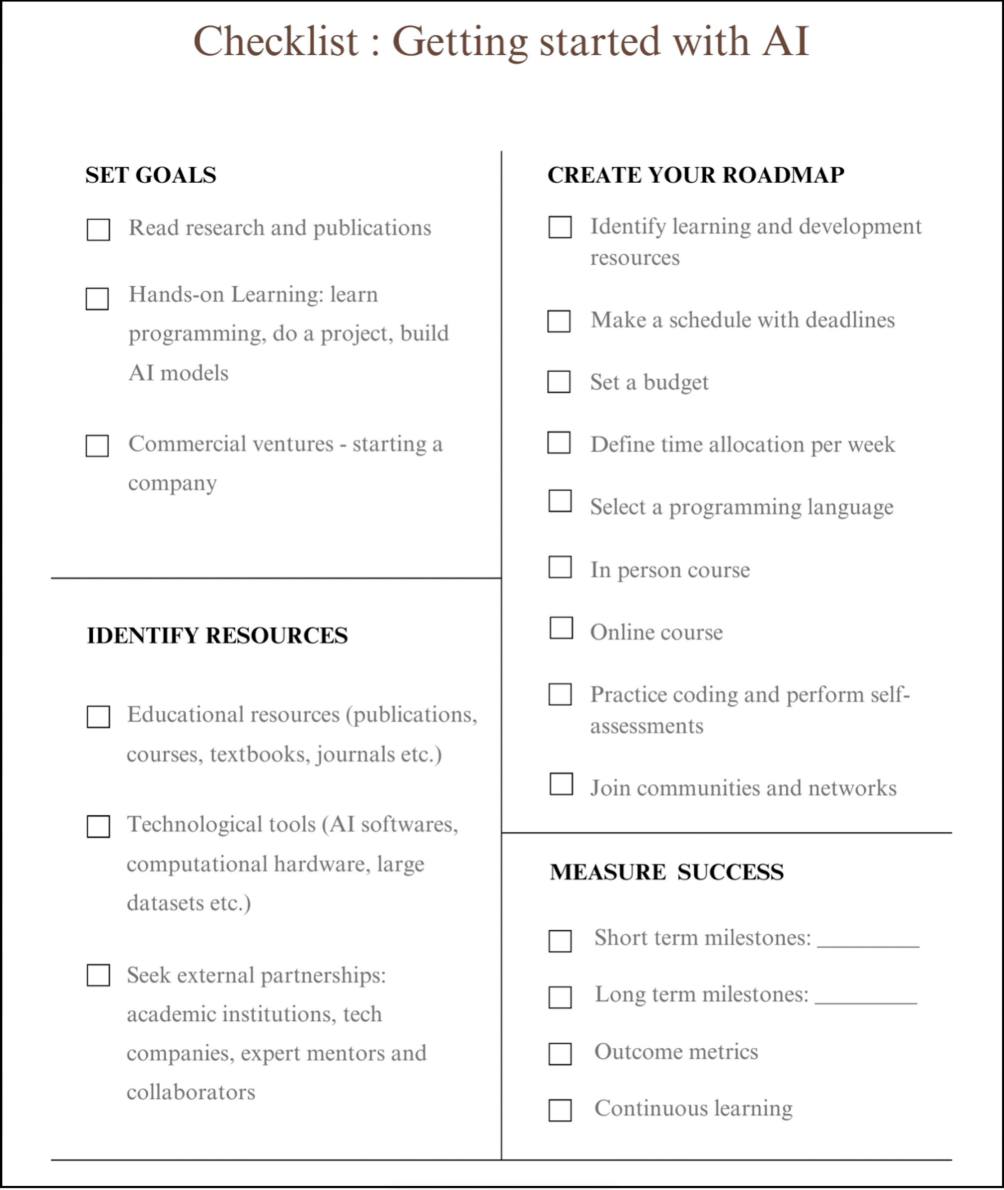
Navigating AI checklist to assist fulfill clinicians’ goals and measure success Image Credits: Hajra Arshad

Some of the process measures could include the successful creation of project protocol, a checklist of resources to follow, institutional review board (IRB) submission of a research proposal, or the creation of an initial team to get started on an innovation pilot (Figure 4). Outcome measures can include the successful completion of courses, the publication of an article, or successful completion of the development of an AI prototype. Similar to any other lifelong learning processes, continuous learning, project trials, or implementation of developed solutions is crucial. Learning from failures, adapting to the project needs, or changing the roadmap is part of the journey toward successful outcomes.

Illustrative uses of cases

Through these illustrative examples, we showcase how navigating AI can be used by clinicians to fulfill personalized AI goals.

Jane, a medical student in her third year, comes across the research on the utility of AI in medicine. She is intrigued by the wide potential of AI and wants to learn more about it. She uses the navigating AI checklist and learning resources listed in the paper to enhance her knowledge in the field. Based on the guidance and resources from navigating AI, she enrolled herself in an online course about AI in medicine that taught her the basics. She then starts to read up on several papers published in AI-focused journals suggested in Table 3. Following the above steps equipped her with the knowledge and expertise to understand the literature, conduct research, and publish her own papers in the future.

Anya, a nurse informatician, wants to work on a project at his hospital that uses AI on patient data to reduce readmissions. Using the navigating AI checklist (Figure 4), he begins by creating a roadmap that starts with refining his goal and improving his understanding of AI by taking the courses suggested in Table 3. After completing a few introductory courses and building his confidence, he enrolled in a formal programming course to learn Python. The books and data sources recommended in Table 3 assist him in his Python learning journey through hands-on practice. In addition, he collaborates with his hospital's data science team, analyzing patient data to identify those at high risk of readmission. While gradually building expertise in Python, he also utilizes low-code platforms to quickly build and test simple models without extensive coding. Following navigating AI’s recommendations, he attends hands-on seminars and datathons to further improve his programming and data interpretation skills. His hands-on experience enables him to effectively deploy the AI project at his hospital, ultimately enhancing discharge planning and minimizing readmissions.

Dr. Smith wants to develop and commercialize a predictive AI algorithm for sepsis. Using the navigating AI checklist (Figure 4) he creates a roadmap that starts with refining his goal and improving his understanding of AI using courses suggested in Table 3. He constitutes a team with technical expertise in AI algorithm development and business model development to get his company started. Using the recommendations of navigating AI, he participates in various conferences for critical care and datathons to learn about the development and implementation of the AI algorithm in a clinical environment. While his business team develops a milestone-based sustainable business model, he engages clinicians to help with validation trials, usability, and feasibility assessments to get the FDA approval to achieve his goal of clinical deployment.

## Conclusions

AI application in healthcare provides new and exciting opportunities to all healthcare professionals for learning, professional development, and entrepreneurship. Despite differing goals, having a formal and structured approach to successfully learn and incorporate AI in the daily work of healthcare professionals is fundamental. A deeper understanding of the nuances of AI is needed for a meaningful contribution to this novel field of AI in healthcare.

Moreover, informed healthcare professionals collaborating with AI developers can help develop more pragmatic, patient-centered solutions. Proper guidance can empower healthcare professionals to use AI not only to enhance patient care but also to drive innovation within their areas of expertise. Navigating AI provides a structured framework to assist clinicians with a goal-based and resourceful journey, using personalized metrics for success.
